# A Letter from Philadelphia

**Published:** 1861-04

**Authors:** 


### Correspondence.

Messrs. Editors:—The *Pennsylvania College of Dental Surgery* had their "Commencement" on the evening of Feb. 28, 1861, in the presence of a large and brilliant audience. The valedictory—a very appropriate address—by Prof. W. Calvert.

The class numbered *sixty-three*, of which *thirty-six* graduated. In the class were representatives from seventeen States, also from England, Germany, Switzerland, Cuba and Turkey.

The Demonstrators report 567 patients treated during the session, for whom the following operations were performed: 720 gold and 360 tin fillings (consuming about twelve ounces of gold foil); treatment and filling of pulp cavities, 176; extraction of teeth and roots, 3,129. Also, removal of salvary calculi, insertion of pivot teeth, treatment of inflammation of gums, alveolar abscess and irregularities.

In the mechanical department, whole sets of teeth inserted,

14; upper sets, 62; upper sets of block, 4; partial sets, 79; also, irregularity, plates, etc.; whole number of teeth mounted, 2,039.

*The Baltimore College*, I learn from a programme, had its twenty-first annual Commencement on the evening of Feb. 26, 1861. The class numbered *fifty-eight*, from which *twenty-nine* graduated. In the class were representatives from fifteen States, also from England and Prussia.

*Vulcanizers.*—These articles have not been free from the usual consequences of competition as a cheapening process, their substantiality decreasing with the decrease of price.

I have just been to see a member of the fraternity, who, some five days ago, had one of these appliances of Vulcan blow up, and that he is now alive is owing solely to the fact that his head was not in a line with the flying top of the machinery; as it is, however, his sight is very materially affected from a portion of the steam, and perhaps the packing, being driven in his face. There are hopes now that he will recover his sight, but at first there were many fears that the sight would be lost.

I examined the wreck, and found that the boiler was made of copper, and the head or cap of cast brass, which screwed on a brass rim attached to the outer surface of the boiler. In the top was what purported to be a fusible metal plug, but which plug remained firm and unaffected. There was also a small metal plug in the side of the upright, and just below the bulb of the thermometer, intended for a safety valve, but which was useless in that position. The fracture occurred in this cast brass head, which blew off, making a clear short fracture all around and in a perpendicular line with the inner surface of the boiler. The brass at no place where fractured was over one-eighth of an inch in thickness, and had the appearance of a very coarse quality of metal.

I was informed that at the time the explosion occurred, the thermometer indicated a heat of 340°, and that he was just about leaning over to turn off the gas. To my question why he ran it to so high a heat, he answered that the manufacturer had assured him that all his machines were thoroughly tested up to a heat of 400°, and that they were entirely safe up to that point.

Now, what is a little peculiar, a similar machine by the same maker (in New York city) exploded in the same manner in same person's work-shop, but a few weeks previously, but fortunately, injured no one. On that occasion the heat was inside of 300°.

I have related these facts that the profession may see that there is necessity for considerable caution in the purchase and employment of these instruments, and that cheap goods of any kind are not always the best, but generally the reverse.

Another case I will mention, which has just come to my knowledge, where, but for thoughtfulness on the part of the operator, injury might have resulted, even with the best appliance. It was the discovery, after waiting the proper time for the heat to come up, that the bulb of the thermometer was fractured, allowing the admission of air, which of course rendered it useless. Had the thermometer been the sole reliance in this case, the result may be conjectured.

What has become of the Baltimore Journal of Dental Science? The number due last January has not yet made its appearance. Yours, O. U. C.

PHILADELPHIA, March 19, 1861.

